# Silibinin Alleviates Muscle Atrophy Caused by Oxidative Stress Induced by Cisplatin through ERK/FoxO and JNK/FoxO Pathways

**DOI:** 10.1155/2022/5694223

**Published:** 2022-01-20

**Authors:** Meng-yi Chi, Hong Zhang, Ya-xian Wang, Xi-peng Sun, Quan-jun Yang, Cheng Guo

**Affiliations:** ^1^School of Pharmacy, Shanghai University of Traditional Chinese Medicine, Shanghai 201203, China; ^2^Department of Pharmacy, Shanghai Jiao Tong University Affiliated Shanghai Sixth People's Hospital, Shanghai 200233, China; ^3^School of Medicine, Shanghai Jiao Tong University, Shanghai 200240, China

## Abstract

Cisplatin (DDP), a widely used chemotherapeutic drug in cancer treatment, causes oxidative stress, resulting in cancer cachexia and skeletal muscle atrophy. This study investigated the effects and activity of silibinin (SLI) in reducing DDP-induced oxidative stress and skeletal muscle atrophy in vivo and in vitro. SLI alleviated weight loss, food intake, muscle wasting, adipose tissue depletion, and organ weight reduction induced by DDP and improved the reduction of grip force caused by DDP. SLI can attenuated the increase in reactive oxygen species (ROS) levels, the decrease in Nrf2 expression, the decrease in the fiber cross-sectional area, and changes in fiber type induced by DDP. SLI regulated the ERK/FoxO and JNK/FoxO pathways by downregulating the abnormal increase in ROS and Nrf2 expression in DDP-treated skeletal muscle and C2C12 myotube cells. Further, SLI inhibited the upregulation of MAFbx and Mstn, the downregulation of MyHC and MyoG, the increase in protein degradation, and the decrease of protein synthesis. The protective effects of SLI were reversed by cotreatment with JNK agonists and ERK inhibitors. These results suggest that SLI can reduce DDP-induced skeletal muscle atrophy by reducing oxidative stress and regulating ERK/FoxO and JNK/FoxO pathways.

## 1. Introduction

Chemotherapy has been used to treat cancer since the 1970s [1, 2] and several recent studies have shown that during treatment with chemotherapy drugs, patients will experience cancer cachexia [3, 4]. As an adverse reaction secondary to cancer, the main manifestations of cancer cachexia are weight loss, muscle weight reduction (i.e., muscle loss), and fat weight reduction, which is often associated with a poor prognosis [5, 6]. Many experimental studies have confirmed that chemotherapeutic drugs can induce cachexia. Mice treated with carboplatin showed bone loss, muscle atrophy, and weakness [7]. The combination of oxaliplatin and 5-fluorouracil also causes muscle atrophy, decreased protein synthesis, autophagy, and mitochondrial changes in mice [8]. Cisplatin (DDP) has also been shown to cause cancer cachexia in mice, characterized by weight loss, muscle mass loss, and activation of the ubiquitin protease pathway [9–11]. DDP-induced cancer cachexia mainly involves the ubiquitin proteasome pathway, autophagy, calcium homeostasis, mitochondrial biogenesis and kinetics, oxidative stress, proinflammatory cytokines, and lipid metabolism [12]. Nowadays, cisplatin is commonly used to induce cachexia in animal models [12, 13].

The ubiquitin proteasome pathway plays a dominant role in the occurrence and development of cancer cachexia. When activated, the expression of muscle atrophy F-box (MAFbx/FbxO32) in the muscle tissues increases, resulting in increased degradation of skeletal muscle [14]. Meanwhile, myostatin (Mstn) is upregulated and myogenesis related to the myosin heavy chain (MyHC), myogenic differentiation antigen (MyoD), and myogenin (MyoG) were downregulated [15–17]. The increase in MAFbx is associated with forkhead box protein O3*α* (FoxO3*α*). FoxO3*α* migrates to the nucleus to activate atrophy and autophagy-related genes by dephosphorylation, thus promoting autophagy formation and muscle atrophy [12, 18]. DDP has been confirmed to cause animal muscle atrophy or C2C12 myotube cell atrophy through autophagy [19, 20]. Oxidative stress has been described as a major factor in the development and occurrence of cachexia. Oxidative damage can indirectly lead to muscle atrophy through redox signals in the degradation pathway or can lead directly to muscle atrophy [21].

Oxidative stress defines a state of imbalance between oxidation and antioxidation mechanisms in the body, which leans towards higher oxidative status, neutrophil inflammatory infiltration, increased protease secretion, and the production of many oxidative intermediates. Oxidative stress is a negative effect produced by free radicals in the body. Oxygen-derived free radicals and their derivatives are collectively referred to as reactive oxygen species (ROS), which include superoxide anion (O_2_^−^), hydroxyl radical (−OH), and hydrogen peroxide (H_2_O_2_). ROS can induce phosphorylation of c-Jun N-terminal kinase (JNK), extracellular signal-regulated kinase 1/2 (ERK1/2), and p38 mitogen-activated protein kinase (p38 MAPK), which are members of the mitogen-activated protein kinase (MAPK) family of serine/threonine kinases. The MAPK family is often involved in cell apoptosis [22, 23]. Furthermore, JNK/ERK related to oxidative stress has been reported to inhibit the nuclear factor erythrocyte 2-related factor 2 transcription factor (Nrf2) during apoptosis [24]. Nrf2 plays an important role in the activation of antioxidant enzymes and regulates the production of intracellular ROS. Under disease conditions, the expression of antioxidant proteins regulated by Nrf2 and Nrf2 decreases [25], which will not protect the body from excessive ROS under oxidative stress. ROS can not only stimulate muscle fiber decomposition but also interfere with anabolism and reduces protein production. Akt/mTOR (protein kinase B/mammalian target of rapamycin) is the main pathway involved in protein synthesis and the regulation of skeletal muscle quality. When the ROS levels in the body are at low levels, this pathway can be activated. In turn, high ROS levels will inhibit the pathway and, thus, inhibit protein synthesis [26]. Oxidative damage caused by free radical formation is one of the causes of cancer cachexia and body damage induced by DDP [12]. DDP significantly increases sirtuin1 (SIRT1) and peroxisome proliferator-activated receptor-*γ* coactivator-1*α* (PGC-1*α*) levels in muscle, fat, and liver tissues of C57BL/6 mice and influences mitochondrial homeostasis [27, 28]. DDP can reduce Nrf2 expression, increase the level of reduced coenzyme II (NADPH) oxidase 4 (NOX4) expression, and increase ROS levels, leading to mitochondrial dysfunction and damage to the body [28]. Many studies have shown that drugs with antioxidant and free radical scavenging activities, such as *Scutellaria baicalensis* and quercetin, can prevent and treat DDP-induced oxidative damage [13, 29, 30]. Altogether, these findings suggest that it is possible to treat cancer cachexia caused by DDP by improving oxidative stress.

Silibinin (SLI) is a natural flavonoid isolated from the silymarin seed extract (*Silybum marianum* (L.) Gaertn.). SLI has antioxidant, immunomodulatory, antifibrotic, antitumor proliferation, and antiviral activities [31, 32]. Silymarin fruits and seeds have been used to treat hepatobiliary-related diseases for more than 2000 years [33] and are often used to treat chronic liver diseases, such as hepatitis and liver cirrhosis [34]. SLI can improve the distribution of fatty acids in lipid droplets, improve liver fat metabolism, and decrease the activation of nuclear factor kappa-B (NF-*κ*B) in the liver [35–37]. In addition, it protects the liver by improving mitochondrial oxidation and reducing oxidative stress by reducing ROS levels, lipid peroxidation levels, and catalase activity [34, 38].

SLI can also treat diabetic tissue lesions and heart disease by reducing oxidative stress. Studies have shown that SLI can restore structural changes of the pancreas in the streptozotocin- (STZ-) induced diabetic rat model, reduce the concentration of malondialdehyde (MDA), increase the concentration of glutathione (GSH), and increase the activity of superoxide dismutase (SOD) and catalase (CAT) [39]. SLI protects rat cardiomyocytes treated with isoproterenol by regulating SIRT1, significantly reduces the release of lactate dehydrogenase (LDH) and the production of MDA, increases SOD activity, increases B-cell lymphoma-2 (Bcl-2) expression, and reduces cytochrome C expression, to protect rat cardiomyocytes from necrosis [40]. SLI has a good antioxidant properties.

SLI may be potentially active in reducing the toxic and side effects of cisplatin without affecting its antitumor activity. SLI has not been reported to alleviate cachexia caused by chemotherapy. The aim of this study was at exploring the effects of SLI on DDP-induced cachexia and its mechanism of alleviating skeletal muscle atrophy, at providing experimental evidence supporting the antioxidant effect of SLI, and at providing a rationale for its development as a drug to alleviate cancer cachexia and skeletal muscle atrophy caused by chemotherapeutic agents such as cisplatin.

## 2. Materials and Methods

### 2.1. Animals

Four-week-old C57BL/6 male mice were purchased from SLAC Experimental Animals Co. Ltd. (Shanghai, China). Mice were kept at a constant temperature and humidity, given a day/night cycle of 12 : 12 h, fed and drank freely, adapted to the environment, and fed a normal diet for one week. All animal experiments were performed in compliance with the ethical requirements of the Laboratory Animal Research Center, Shanghai Jiao Tong University Affiliated Shanghai Sixth People's Hospital. The experimental protocol was approved by the Animal Welfare Ethics Committee of Shanghai Sixth People's Hospital (no: DWLL2021-0752).

### 2.2. DDP-Induced Skeletal Muscle Atrophy Model and SLI Administration Regimen

A total of 25 male C57BL/6 mice aged 5 weeks were randomly divided into 5 groups: normal control group (NC) (normal saline, intraperitoneally (i.p.); 0.3% sodium carboxymethylcellulose (CMC Na), 624R023, Solarbio, China, per os (p.o.)); Lewis lung cancer-bearing group (LLC) (normal saline, i.p.; 0.3% CMC-Na, p.o.), DDP model group (LLC + DDP) (DDP, 86491, MedChem Express, New Jersey, 4 mg/kg/2 d, i.p.; 0.3% CMC-Na, p.o.); SLI low-dose group (LLC + DDP + SLI [L]) (DDP, 4 mg/kg/2 d, i.p.; SLI, SS8540, Solarbio, China, 40 mg/kg/d, p.o., 0.3% CMC-Na); and SLI high-dose group (LLC + DDP + SLI [H]) (DDP, 4 mg/kg/2 d, i.p.; SLI, 80 mg/kg/d, p.o., 0.3% CMC-Na). On the first day of the experiment, except for the NC group, mice were inoculated with LLC cells (1 × 106 cells/mouse) on the dorsal side of the right forelimb. The weight and food intake of the mice in the five groups was recorded and managed on days 6–15. The recording and management time was 12 : 00–13 : 00 every day. On day 15 of the experiment, the mice were anesthetized after grip strength was measured. Mice were euthanized and tissues were collected. Tumor, gastrocnemius muscle (GA), anterior tibial muscle (TA), and epididymis adipose tissue were collected and weighed. A portion of the GA was labeled and fixed in 4% paraformaldehyde (p1110, Solarbio, China). The remaining GA and other tissues were snap frozen in liquid nitrogen after labeling.

### 2.3. Cell Culture and Administration Scheme

Murine C2C12 myoblasts (GNM26) and LLC lung cancer cells were provided by the stem cell bank of the Chinese Academy of Sciences. Cells were cultured in 5% CO_2_ at 37°C. The C2C12 myoblasts were cultured in Dulbecco's modified Eagle medium (DMEM) (10-013-cv, Corning Life Sciences, New York) supplemented with 10% fetal bovine serum (FBS) (10141, Gibco, Massachusetts) and penicillin streptomycin (100 units/mL) (p1400, Solarbio, China). C2C12 cells were inoculated in a six-well plate at a density of 5 × 10^5^ cells per well and replaced with DMEM medium containing 2% horse serum (16050122, Gibco, Massachusetts) for 6 days to induce C2C12 myoblasts to differentiate into myotube cells. C2C12 myotube cells were treated with 50 *μ*M DDP and SLI of different concentrations for 24 hours. LLC cells were cultured in DMEM supplemented with 10% FBS and penicillin-streptomycin (100 units/mL).

### 2.4. CCK8

C2C12 myoblasts (1 × 10^4^ cells/pores) were inoculated into 96-well culture plates and induced to differentiate into myotubes. LLC cells (1 × 10^4^ cells/pores) were inoculated into a 96-well culture plate. C2C12 myotube cells or LLC cells were treated with different concentrations of SLI for 24 and 48 hours with 6 repeated holes. Cell proliferation was determined using the CCK-8 (Dojindo Molecular Technologies, Kumamoto, Japan) by adding 100 *μ*L CCK-8 reagent (10 *μ*g/mL) to each well. The optical density (OD) was measured at 450 nm by using a plate reader (BioTek, Vermont). Cell proliferation was measured after culturing at 37°C for 1 hour.

### 2.5. Grip Strength Test

The grip strength of the mouse forelimb was measured using a grip meter (Sh-20, NSCING, Nanjing, China). The mouse was placed on the sensor wire mesh, and the mouse tail was dragged in parallel. The maximum force generated when the tail was pulled horizontally away from the wire mesh through the grip strength was recorded. Each mouse was measured 3 times with an interval of 10 s. The average of the three peak forces was taken as the grip strength value and used to evaluate muscle strength.

### 2.6. Histology and Hematoxylin-Eosin Staining

The GA muscle was immobilized in 4% paraformaldehyde and embedded in paraffin. The paraffin blocks were cut into 4 *μ*m thick slices and stained with a hematoxylin-eosin (H&E) reagent box (G1121, Solarbio, China). H&E staining for the cross-sectional area analysis was used for the evaluation of muscle slices, and images were captured using cellSens software (Olympus Corporation, Tokyo, Japan). The fiber cross-sectional area of the GA muscle was obtained from an assessment of 160 fibers.

### 2.7. Fast and Slow Muscle-Type Assay

The GA muscle was immobilized in 4% paraformaldehyde, cut into 10 *μ*m frozen sections at low temperature, and then placed on glass slides. Slow muscle fibers and fast muscle fibers in each GA muscle were determined by mouse monoclonal antibodies against myosin heavy chain MyHC I (ab11083, Abcam) and anti-myosin heavy chain MyHC II (ab51263, Abcam) mouse monoclonal antibodies. After incubation with anti-MyHC I and anti-MyHC II primary antibodies, phosphate-buffered saline (PBS) was used to incubate the sections with the corresponding secondary antibodies and 4′,6-diamidino-2-phenylindole (DAPI) (D9542, Sigma–Aldrich, St. Louis, MO) and all nuclei were incubated at room temperature for 1 hour. Anti-mouse IgG1 Alexa Fluor 488 antibody- (green-) labeled MyHC I and anti-mouse IgG1 Alexa Fluor 594 antibody- (red-) labeled MyHC II were used. Analysis was performed using ImageJ (NIH, Bethesda, MD) to determine the fluorescence intensity of fast and slow muscles. Image analysis was performed in the standardized field of view with the color code, and the maximum fluorescence intensity in the standardized field of view was measured automatically.

### 2.8. Myosin Type I Assay and Myosin Type II Assay

Frozen GA muscle tissue fixed in liquid nitrogen was cut into 10 *μ*m frozen sections at low temperature and stained with the ATPase Staining Kit (G2380, Solarbio, China). Under alkaline conditions, type I muscle fibers were not stained, and type II muscle fibers were deeply stained. Under acidic conditions, type I muscle fibers were deeply stained. Stained sections were used for the CSA analysis. Muscle sections were evaluated using cellSens software. The fiber cross-sectional area was obtained from the evaluation of 80 fibers.

### 2.9. ROS Assay

Dihydroethidium (DHE) and 2′,7′-dichlorodihydrofluorescein diacetate (DCFH-DA) are widely used for the determination of reactive oxygen species [21, 41]. The frozen GA muscle was cut into 5 *μ*m thick sections, placed on glass slides, and stained with 10 *μ*mol/L DHE (D7008, Sigma-Aldrich, St. Louis, Mo). After staining, the slides were cultured in a light-protected and humidified chamber at 37°C for 30 minutes. The slides were then washed twice with PBS and sealed with a cover glass. Finally, the fluorescence of DHE was observed by a fluorescence microscope. ImageJ was used for analysis to determine the fluorescence intensity of DHE-stained cells. Image analysis was carried out in the standardized field of view on the color code, and the maximum fluorescence intensity in the standardized field of view was measured automatically.

C2C12 myotube cells treated with drugs for 24 hours were washed with PBS 3 times, and then, 10 *μ*mol/L DCFH-DA (D6883, Sigma Aldrich, St. Louis, Mo) prepared with serum-free medium was added and the cells were cultured in a 5% CO_2_ environment at 37°C for 30 minutes. Next, cells were washed with PBS 3 times, and the fluorescence of DCFH-DA (excitation wavelength 488 nm, emission wavelength 530 nm) was observed with a fluorescence microscope.

### 2.10. Immunoblotting

C2C12 myotube cells were fixed with 4% PBS-buffered paraformaldehyde at room temperature for 15 minutes and then infiltrated with 0.5% Triton X-100 for 20 minutes. C2C12 myotube cells were blocked with 5% bovine serum albumin at room temperature for 1 hour, then, incubated with anti-MYH primary antibody (sc-376157, Santa Cruz Biotechnology) overnight at 4°C, incubated with mouse Alexa Fluor®488 conjugate (4408, Cell Signaling Technology, Massachusetts) antibody for 1 hour, and then, incubated with 10 *μ*g/mL DAPI for 5 minutes. Wash with PBS 3 times between operations for 5 minutes each time. Analyze the photos using cellSens software.

### 2.11. Determination of the Reduced (GSH) and Oxidized (GSSG) Glutathione Content

GSH and GSSG content was determined using a total glutathione/oxidized glutathione assay kit (a061-1, Nanjing Jiancheng Institute of Bioengineering, China). Muscle tissues of the same size were obtained from each group and protein concentration was measured. According to the manufacturer's instructions, the reagents and samples were added in sequence and the 96-well plate was shaken before being placed in the microplate reader. The absorbance value A1 at 405 nm was read for 30 seconds and allowed to stand at room temperature (25°C) for 10 minutes, and then, the absorbance value A2 at 405 nm was read for 10 minutes and 30 seconds. The contents of T-GSH, GSSG, and reduced-GSH were determined according to the formula provided by the manufacturer.

### 2.12. Western Blot Analysis

GA muscle and C2C12 myotube cells were lysed in RIPA buffer (R0010, Solarbio, China) containing a mixture of protease and phosphatase inhibitors (P002, NCM Biotechnology, China) according to the manufacturer's instructions. Nuclear protein was extracted using the Nuclear Protein Extraction Kit (R0050, Solarbio, China). The lysate was centrifuged at 14,000 rpm at 4°C for 15 minutes. The supernatant was retrieved and protein concentrations were evaluated using the double cinchonic acid protein analysis kit (P0011, Beyotime, China). The proteins were denatured in 5x loading buffer (l LT101S, Epizyme, China). A total of 30 *μ*g protein was loaded and separated by 10% sodium sulfonate polyacrylamide gel, and then, proteins were transferred to nitrocellulose membranes (Millipore Corporation, Bedford, MA). After blocking in PBS containing 5% BSA at room temperature for 1 hour, the membrane was exposed to the primary antibody at 4°C overnight and then incubated with IR-Dye680-conjugated anti-mouse or anti-rabbit secondary antibody (926-68071, LI-COR Biosciences, Nebraska) (dilution: 1 : 5000) at room temperature for 1 hour. The primary antibodies (dilution: 1 : 1000) used in the study were the following: anti-MYH (B-5) (sc-376157, Santa Cruz Biotechnology, California), anti-Atrogin1/MAFbx (ab168372, Abcam, Massachusetts), anti-FoxO3*α* (#12829, Cell Signaling Technology, Massachusetts), anti-p-SAPK/JNK (#9255, Cell Signaling Technology, Massachusetts), anti-SAPK/JNK (#9252, Cell Signaling Technology, Massachusetts), anti-p-ERK1/2 (#4370, Cell Signaling Technology, Massachusetts), anti-ERK1/2 (#4695, Cell Signaling Technology, Massachusetts), anti-epidermal growth factor receptor (EGFR) (#4267, Cell Signaling Technology, Massachusetts), anti-Ras (#3339, Cell Signaling Technology, Massachusetts), anti-tubulin (#2128, Cell Signaling Technology, Massachusetts), anti-Lamin A/C (#2032, Cell Signaling Technology, Massachusetts), anti-MyoG (ab1835, Abcam, Massachusetts), anti-MyoD (ab126726, Abcam, MA, United States), anti-Myostatin/GDF8 (ab203076, Abcam, MA, United States), anti-Nrf2 (A1244, ABclonal, China), and anti-c-FOS (A17351, ABclonal, China). Tubulin or Lamin A was used as an internal control.

### 2.13. RNA Extraction and Reverse Transcription Quantitative PCR (RT-qPCR)

Total RNA was isolated from GA muscle using RNAsio Plus (9109 TaKaRa, Kyoto, Japan) according to the manufacturer's instructions. Complementary DNA (cDNA) was synthesized from 2000 ng of total RNA with HiScript II Q Select RT SuperMix (R223-01, Vazyme, Nanjing, China). Quantitative real-time polymerase chain reaction (qRT-PCR) was performed using a reaction mixture containing the SYBR Green Master Mix (Q711-02-AA, Vazyme, Nanjing, China). Results of the RPS18 gene standardized 2^−ΔΔ*CT*^ expression was calculated by the relative quantitative method. The sequences of primer pairs are shown in Table 1.

### 2.14. Statistical Analysis

GraphPad Prism V8.3.0 (GraphPad Software Inc., San Diego, California) was used for statistical analysis. Data were expressed as mean ± standard error of the mean (SEM) or standard deviation (SD). A *p* value < 0.05 was considered statistically significant. To compare the results of the two groups, the *t*-test was carried out. To compare more measurements, one-way or two-way ANOVA was performed.

## 3. Result

### 3.1. SLI Alleviated the Cachexia of LLC Tumor-Bearing Mice Induced by Cisplatin

**Figure 1:**
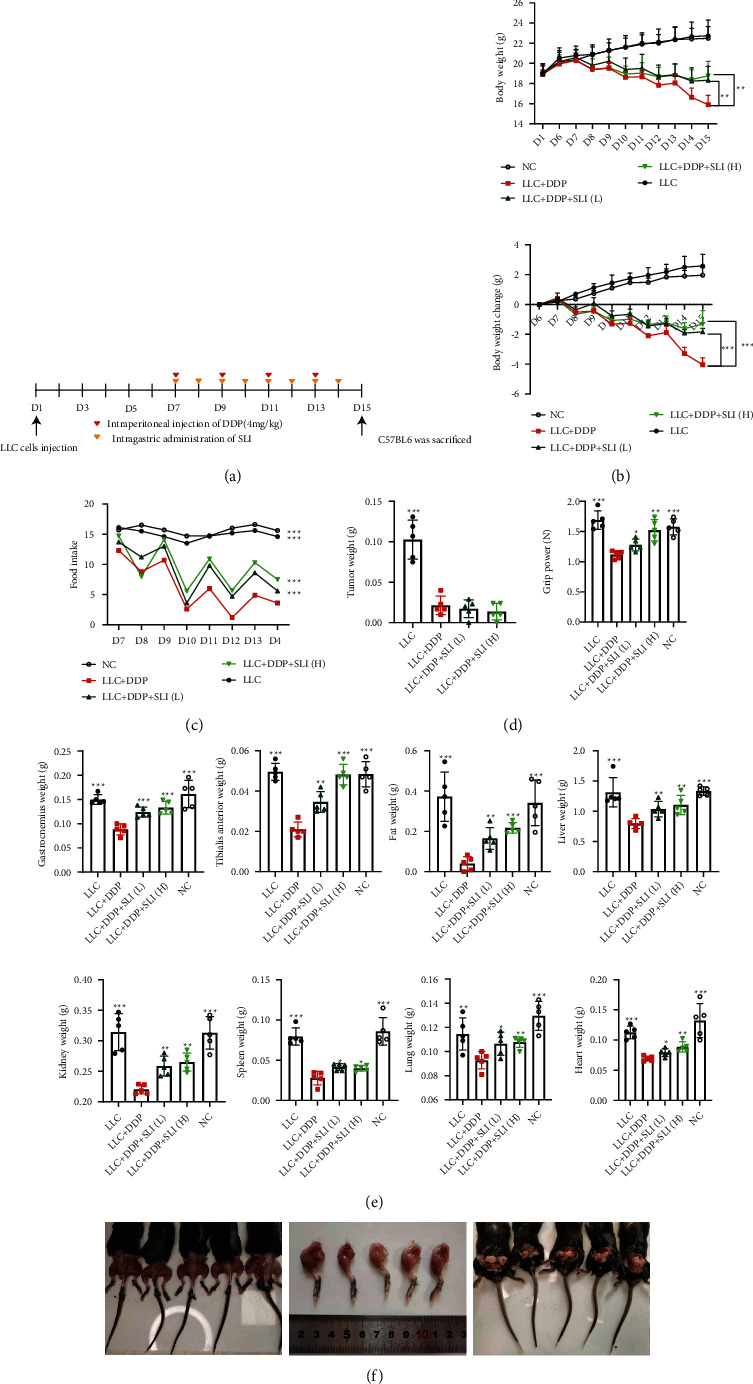
SLI alleviated the weight loss of the body, food intake, muscle, epididymal adipose, liver, kidney, spleen, lung, and heart and weakened the forelimb grip strength of tumor-bearing mice after DDP treatment and did not affect the antitumor activity of DDP. (a) 5-week-old C57BL/6 mice were treated with DDP (4 mg/kg/2 d, i.p.) and given high and low doses of SLI treatment (80/40 mg/kg/d, p.o.) simultaneously; the treatment lasted for 8 days. (b) The weight of the mice was measured on day 1 and days 6–15. The change in body weight was relative to the first administration (day 6 of tumor bearing). Data are shown as mean ± SD, *n* = 50/group; ^∗∗^*p* < 0.01, ^∗∗∗^*p* < 0.001 versus group LLC + DDP. (c) Total daily food intake of mice in each group. Data are shown as an individual values, *n* = 5/group; ^∗∗∗^*p* < 0.001 versus group LLC + DDP. (d) Tumor weight was measured after the mice were euthanized, and grip strength of the forelimbs was measured before the mice were sacrificed. Data are shown as mean ± SD, *n* = 5/group; ^∗^*p* < 0.05, ^∗∗^*p* < 0.01, ^∗∗∗^*p* < 0.001 versus group LLC + DDP. (e) After the mouse was euthanized, the GA muscle, TA muscle, epididymis adipose, liver, kidney, spleen, lung, and heart were removed and weighed. Data are shown as mean ± SEM, *n* = 5/group; ^∗^*p* < 0.05, ^∗∗^*p* < 0.01, ^∗∗∗^*p* < 0.001 versus group LLC + DDP. (f) Comparison of leg muscles and epididymal adipose of typical mice in each group, from left to right, LLC, LLC + DDP, LLC + DDP + SLI (L), LLC + DDP + SLI (H), and NC groups, respectively.

To explore the effects of SLI on LLC tumor-bearing mice treated with DDP, five-week-old C57BL/6 mice were treated with DDP (4 mg/kg/2 d, i.p.) 4 times at day 7 of tumor bearing. The SLI group was treated with SLI (80/40 mg/kg/d, p.o.) continuously for 8 days (Figure 1(a)). SLI reduced DDP-induced weight loss in a dose-dependent manner (Figure 1(b)). SLI slowed the decrease in food intake in mice after DDP, and the effect was dose dependent (Figure 1(c)). SLI reduced the decrease in grip strength caused by DDP and the tumor size of SLI-treated mice while DDP treatment did not differ significantly from that of the LLC + DDP group, albeit tumor sizes were smaller than those of the LLC group (Figure 1(d)). As shown in Figure 1(e), SLI reduced the weight loss of GA muscle, TA muscle, epididymis adipose, liver, kidney, spleen, lung, and heart caused by DDP. The comparison between leg muscles and epididymal fat of typical mice in each group is shown in Figure 1(f). From left to right, we have the following: LLC, LLC + DDP, LLC + DDP + SLI (L), LLC + DDP + SLI (H), and NC group mouse, respectively. The LLC + DDP group mouse showed marked leg muscle atrophy and reduction in epididymal fat compared to mice in other groups, evidencing cachexia from cancer, which was improved by treatment with SLI.

### 3.2. SLI Alleviated Muscle Fiber Atrophy in DDP-Treated LLC Tumor-Bearing Mice

**Figure 2:**
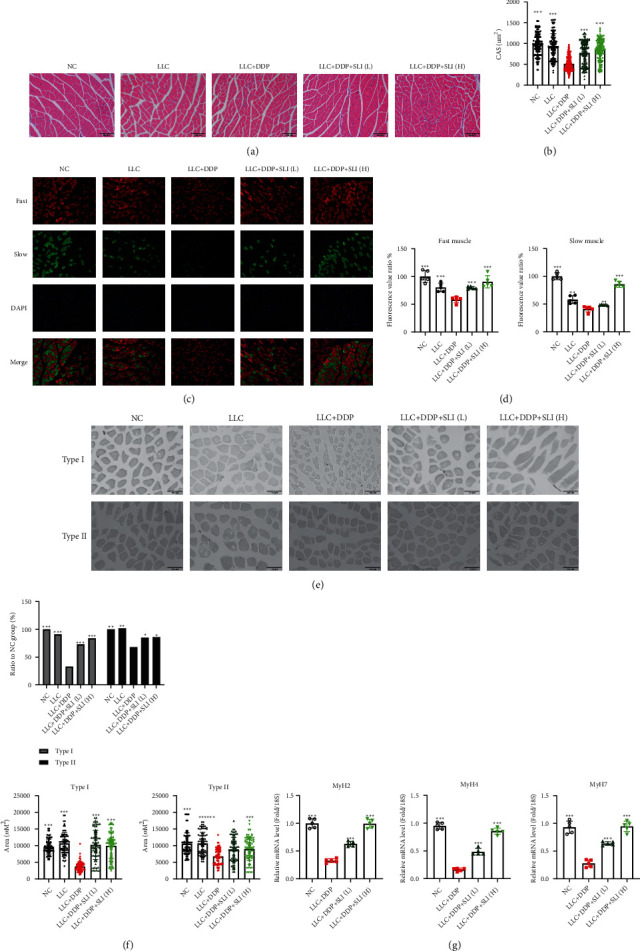
Effects of SLI treatment on muscle fiber CSA and muscle fiber types in tumor-bearing mice after DDP treatment. (a) H&E staining of mouse GA muscle sections (scale bar = 100 *μ*m). (b) CSA was measured using cellSens software, and the mean CSA is represented. Data are shown as mean ± SD, *n* = 160/group; ^∗∗∗^*p* < 0.001 versus group LLC + DDP. (c) Section stained with MyHC I and MyHC II of mouse GA muscle and observed under fluorescence microscope (scale bar = 100 *μ*m). (d) The changes of fast muscle fibers (MyHC II) and slow muscle fibers (MyHC I) were analyzed by ImageJ. Data are shown as mean ± SD, *n* = 5/group; ^∗∗^*p* < 0.01, ^∗∗∗^*p* < 0.001 versus group LLC + DDP. (e) Section stained with ATPase from mouse GA muscle (scale bar = 100 *μ*m). (f) Changes of type I and type II myosin. Data are shown as mean ± SD, *n* = 80/group; ^∗^*p* < 0.05, ^∗∗^*p* < 0.01, ^∗∗∗^*p* < 0.001 versus group LLC + DDP. (g) qRT-PCR was used to detect the expression of MYH2, MYH4, and MYH7 mRNA in the GA muscle; RPS18 was used as an internal control. Data are shown as mean ± SD, *n* = 5/group; ^∗^*p* < 0.05, ^∗∗^*p* < 0.01, ^∗∗∗^*p* < 0.001 versus group LLC + DDP.

We evaluated the effects of DDP and DDP + SLI treatment on muscle fibers in mice. CSA of the muscle fiber was analyzed after H&E staining of GA muscle. As shown in Figures 2(a) and 2(b), the CSA of GA muscle fibers in the LLC + DDP + SLI groups was significantly higher than that in the LLC + DDP group. SLI also improved the distribution of fiber types in LLC tumor-bearing mice treated with DDP. Skeletal muscle consists mainly of two types of muscle fibers, type I (MyHC I, slow type) and type II (MyHC II, fast type). In this study, both types of muscle fiber decreased after DDP treatment, with type I showing a greater decrease. SLI therapy can reduce changes in the number and type of muscle fibers in a dose-dependent manner (Figures 2(c)–2(f)). Two types of muscle fibers are encoded by different genes, of which MyHC I is encoded by MYH7 and MyHC II by the MYH2 and MYH4 genes. After DDP treatment, MYH2, MYH4, and MYH7 expression decreased, with MYH7 decreasing more significantly. After SLI treatment, the expression of all three genes increased in a dose-dependent manner (Figure 2(g)).

### 3.3. SLI Alleviated Oxidative Stress in the Muscle of LLC Tumor-Bearing Mice Treated with DDP

**Figure 3:**
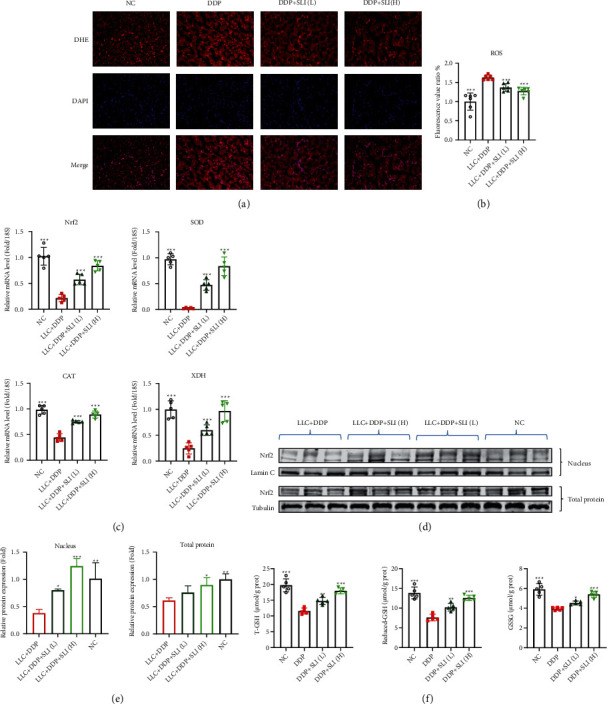
Effect of SLI treatment on the muscle ROS content in tumor-bearing mice after DDP treatment. (a) GA muscle sections of mice were stained with DHE under a fluorescence microscope (scale = 100 *μ*m). (b) Relative fluorescence intensity with DHE stain. Data are shown as mean ± SD, *n* = 5/group; ^∗∗∗^*p* < 0.001 versus group LLC + DDP. (c) qRT-PCR was used to detect the expression of Nrf2, SOD, CAT, and XDH mRNA in the GA muscle; RPS18 was used as an internal control. Data are shown as mean ± SD, *n* = 5/group; ^∗∗∗^*p* < 0.001 versus group LLC + DDP. (d) Nrf2 expression was detected by Western blotting: total Nrf2 expression using tubulin as the internal control and nuclear Nrf2 expression using Lamin A as the internal control. (e) The relative expression level of the protein was detected by ImageJ software. Tubulin and Lamin A were used as internal controls and corrected to the NC group. Data are shown as mean ± SD, *n* = 3/group; ^∗^*p* < 0.05, ^∗∗^*p* < 0.01, ^∗∗∗^*p* < 0.001 versus group LLC + DDP. (f) Results of the determination of T-GSH, GSSG, and reduced GSH in muscle tissue. Data are shown as mean ± SD, *n* = 3/group; ^∗^*p* < 0.05, ^∗∗^*p* < 0.01, ^∗∗∗^*p* < 0.001 versus group LLC + DDP.

Type I (slow-type) fibers, which contain oxygen, mitochondria, and myoglobin, exhibit higher oxidative metabolism, more mitochondria, and enhanced antioxidant defense [42]. Slow-type fibers, which are more oxidizing, rely on proteasome degradation to block oxidized proteins; a major cause of cancer cachexia is oxidative stress [43, 44]. In oxidative stress, ROS levels increase and antioxidant enzyme activity decreases [45, 46]. We evaluated whether DDP induced excessive ROS production and oxidative stress in the muscles of mice. As shown in Figures 3(a) and 3(b), the DHE binding region was larger in the LLC + DDP group and the fluorescence intensity was higher than that in the normal group. The expression of mRNA of the antioxidant enzymes SOD, CAT, XDH, and Nrf2 decreased significantly in the DDP group and increased in the LLC + DDP + SLI (L) group and the LLC + DDP + SLI (H) group compared to the DDP group (Figure 3(c)). Furthermore, measuring the expression of Nrf2 protein confirmed that SLI improved oxidative stress caused by DDP. As shown in Figures 3(d) and 3(e), the expression of Nrf2 protein in the DDP group decreased significantly regardless of nuclear protein or total protein, while the expression of Nrf2 protein in mice treated with SLI increased and tended to reach the same levels of expression as the normal group. Furthermore, the levels of T-GSH, GSSG, and reduced GSH in the muscle of mice in the DDP group were also negatively regulated and SLI treatment could improve this situation (Figure 3(f)). The results showed that the treatment of SLI alleviated the oxidative stress caused by DDP.

### 3.4. Effects of SLI on the Expression of MyHC, MAFbx, ERK/FoxO Signaling Pathway, and JNK/FoxO Signaling Pathway in GA Muscle

**Figure 4:**
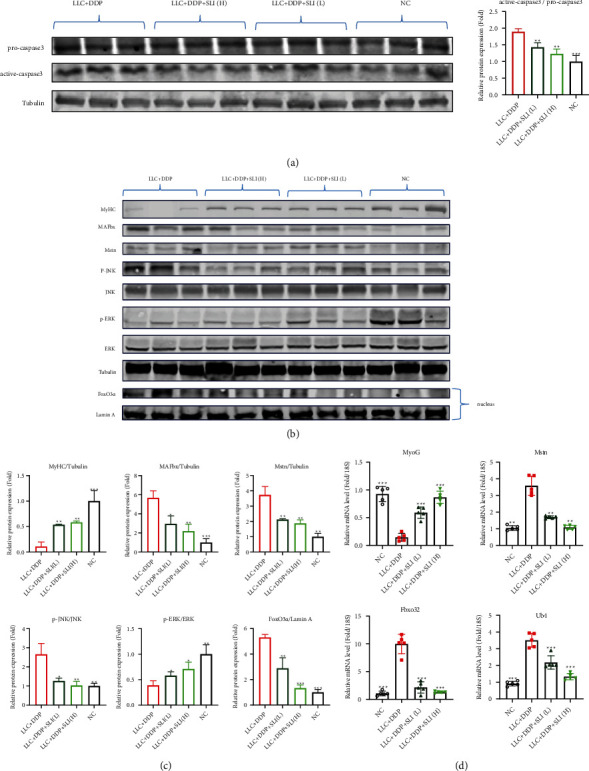
SLI increased MyHC expression in the GA muscle, decreased MAFbx and Mstn expression, and regulated JNK and ERK to inhibit FOXO entry into the nucleus. (a) Using tubulin as an internal control, the expression of procaspase3 and active caspase3 was detected by Western blotting; the relative expression level of protein was detected by ImageJ software. Tubulin was used as internal controls and corrected to the NC group. Data are shown as mean ± SD, *n* = 3/group; ^∗∗^*p* < 0.01, ^∗∗∗^*p* < 0.001 versus group LLC + DDP. (b) Using tubulin as an internal control, the expression of the MyHC, MAFbx, Mstn, JNK/FoxO, and ERK/FoxO signaling pathways was detected by Western blotting; the expression of nuclear FoxO3*α* using Lamin A as an internal control. (c) The relative expression level of protein was detected by ImageJ software. Tubulin and Lamin A were used as internal controls and corrected to the NC group. Data are shown as mean ± SD, *n* = 3/group; ^∗^*p* < 0.05, ^∗∗^*p* < 0.01, ^∗∗∗^*p* < 0.001 versus group LLC + DDP. (d) The qRT-PCR method to detect the expression of MyoG, Mstn, Fbxo32, and Ub mRNA in the GA muscle; RPS18 was used as an internal control. Data are shown as mean ± SD, *n* = 5/group; ^∗∗^*p* < 0.01, ^∗∗∗^*p* < 0.001 versus group LLC + DDP.

The activation of protein degradation-related pathways is the cause of muscle atrophy in cachexia. Skeletal muscle degradation is usually accompanied by the activation of the ubiquitin system, upregulation of MAFbx, and downregulation of MyHC expression in muscle-specific E-3 ubiquitin ligase [16]. The expression of active caspase3 in mice was upregulated after cisplatin, indicating the occurrence of muscle apoptosis, which could be improved by SLI treatment (Figure 4(a)). In addition, after SLI treatment, the expression of MAFbx protein related to muscle degradation was significantly downregulated and the expression of MyHC and MyoG protein was significantly upregulated (Figures 4(b) and 4(c)). Meanwhile, the mRNA expression of MAFbx (Fbxo), ubiquitin, MyHC, and MyoG was restored to the same level as that of the normal control group after SLI treatment (Figure 4(d)).

ERK and JNK are two main members of the MAPK family involved in cell proliferation, differentiation, and apoptosis. ROS can activate JNK phosphorylation and downregulate ERK phosphorylation, triggering cell apoptosis [22]. Western blotting was used to detect ERK and JNK phosphorylation after DDP and SLI treatment. As shown in Figures 4(a) and 4(b), DDP significantly inhibited ERK1/2 activation and increased JNK activation, after SLI treatment, while ERK phosphorylation was upregulated and JNK phosphorylation was downregulated. The phosphorylation of JNK and ERK influenced FoxO3 expression [47, 48], and FoxO3 inhibited muscle cell differentiation and muscle atrophy under stress, which was blocked by SLI treatment (Figures 4(b) and 4(c)).

### 3.5. SLI Protected C2C12 Myotube Cells after DDP through the ERK/FoxO Signaling Pathway and JNK/FoxO Signaling Pathway

**Figure 5:**
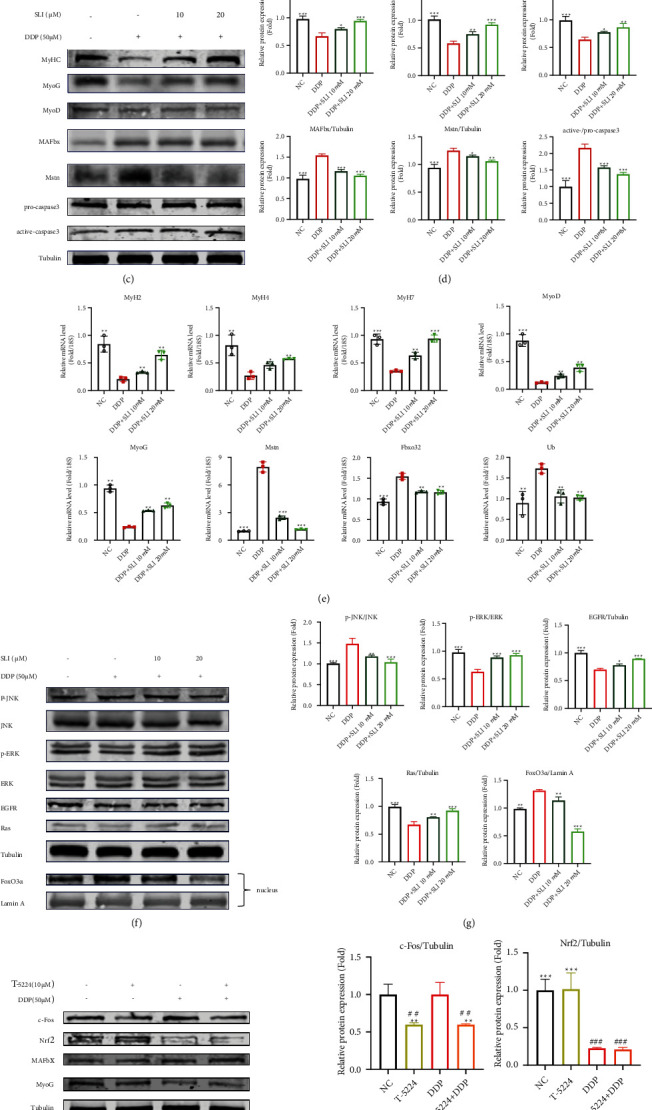
Effects of SLI on C2C12 myotube differentiation and degradation and ERK/FoxO and JNK/FoxO pathways under DDP treatment. (a) SLI prevents DDP-induced differentiated C2C12 myotube atrophy (scale = 100 *μ*m). (b) The fusion indices of C2C12 myotube cells differentiated by MyHC were measured by ImageJ software. Data are shown as mean ± SD, *n* = 3/group; ^∗∗∗^*p* < 0.001 versus group DDP. The area indices of the differentiated C2C12 myotube cells differentiated by MyHC were measured by ImageJ software. Data are shown as mean ± SD, *n* = 30/group; ^∗∗∗^*p* < 0.001 versus group DDP. (c) Using tubulin as an internal control, the expression of MyHC, MyoG, MyoD, MAFbx, Mstn, procaspase3, and active caspase3 was detected by Western blotting; the expression of nuclear FoxO3*α* using Lamin A as the internal control. (d) The relative expression levels of MyHC, MyoG, MyoD, MAFbx, Mstn, procaspase3, and active caspase3 were detected by ImageJ software. Tubulin was used as the internal controls and corrected to the untreated group. Data are shown as mean ± SD, *n* = 3/group; ^∗^*p* < 0.05, ^∗∗^*p* < 0.01, ^∗∗∗^*p* < 0.001 versus group DDP. (e) The qRT-PCR method to detect the expression of MYH2, MYH4, MYH7, MyoD, MyoG, Mstn, Fbxo32, and Ub mRNA in the GA muscle; RPS18 was used as an internal control. Data are shown as mean ± SD, *n* = 3/group; ^∗∗^*p* < 0.01, ^∗∗∗^*p* < 0.001 versus group DDP. (f) Using tubulin as an internal control, the expression of the JNK/FoxO and ERK/FoxO signaling pathways was detected by Western blotting; the expression of nuclear FoxO3*α* using Lamin A as the internal control. (g) Relative expression levels of proteins related to the JNK/FoxO and ERK/FoxO signal pathways were detected by ImageJ software. Tubulin and Lamin A were used as internal controls and corrected to the untreated group. Data are shown as mean ± SD, *n* = 3/group; ^∗^*p* < 0.05, ^∗∗^*p* < 0.01, ^∗∗∗^*p* < 0.001 versus group DDP. (h) Using tubulin as an internal control, the expression of c-Fos, MAFbx, and MyoG was detected by Western blotting; the relative expression levels of c-Fos, MAFbx, and MyoG were detected by ImageJ software. Tubulin was used as the internal controls and corrected to the untreated group. Data are shown as mean ± SD, *n* = 3/group; ^∗^*p* < 0.05, ^∗∗^*p* < 0.01, ^∗∗∗^*p* < 0.001 versus group DDP; ^###^*p* < 0.001 versus group NC.

After 24 and 48 hours of SLI treatment, the drug showed some killing effect on LLC cells and modest effects on C2C12 myotube cells at high dose (Figure S1). The 24-hour intervention showed that exposure to SLI at 10 *μ*M and 20 *μ*M could promote the differentiation of C2C12 myotube cells in the normal state (Figure S2). SLI of 10 *μ*M and 20 *μ*M could prevent DDP-induced atrophy of C2C12 myotube cells within 24 hours (Figures 5(a) and 5(b)). MyoG and MyoD are positively correlated with myotubular cell differentiation [17]. SLI can upregulate protein and mRNA expression of MyHC, MyoG, and MyoD and downregulate the protein and mRNA expression of Mstn and MAFbx (Figures 5(c)–5(e)); the downregulation of ERK phosphorylation and upregulation of JNK phosphorylation in C2C12 myotube cells resulted in the incorporation of FoxO3 into the nucleus (Figures 5(f) and 5(g)). Because the JNK pathway is related to the AP-1 pathway [49], we verified the effects of the AP-1 pathway on C2C12 myotube cells. The AP-1 pathway inhibitor T-5224 was applied to C2C12 myotube cells, and the expression of c-Fos was detected to verify the efficacy of the inhibitor [50] in the NC group, T-5224 group, DDP + T-5224 group, and DDP groups. As shown in Figure 5(h), compared with that of the normal control group, after T-5224 treatment 24 hours, the expression of c-Fos decreased in C2C12 myotube cells but there was no significant change in the expression of Nrf2 and atrophy-related proteins MAFbx and MyoG. Meanwhile, compared with that of the DDP group, the expression of Nrf2, MAFbx, and MyoG changed significantly. There were no significant differences between the expression of Nrf2, MAFbx, and MyoG in the DDP + T-5224 group and the DDP group, indicating that the AP-1 pathway had no significant relationship with DDP-induced C2C12 myotube cell atrophy. Therefore, it is judged that the AP-1 pathway is not involved in DDP-induced oxidative stress and cancer cachexia.

### 3.6. SLI Alleviated Oxidative Stress Induced by DDP in C2C12 Myotube Cells

**Figure 6:**
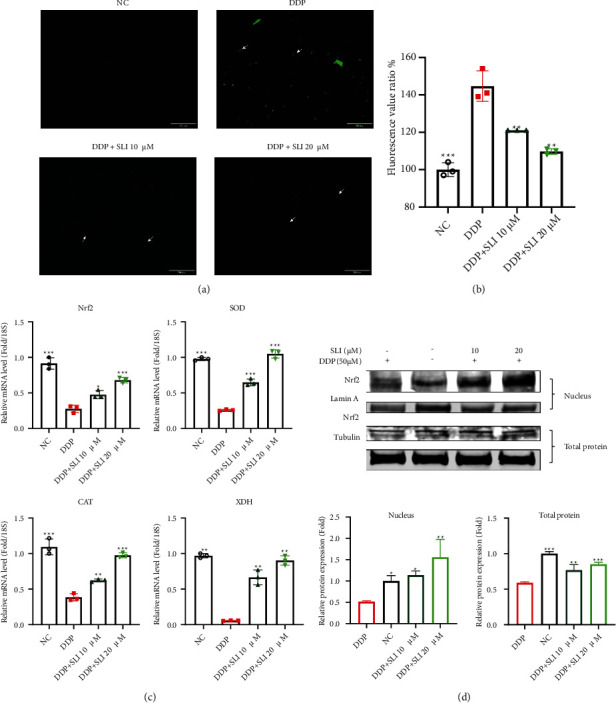
Effect of SLI treatment on the ROS content of C2C12 myotube cells after DDP. (a) C2C12 myotube cells were stained with DCFH-DA and observed under a fluorescence microscope (scale = 500 *μ*m). The green fluorescence indicated by the white arrow is ROS combined with DCFH-DA. (b) Relative fluorescence intensity of DCFH-DA staining. Data are shown as mean ± SD, *n* = 3/group; ^∗∗^*p* < 0.01, ^∗∗∗^*p* < 0.001 versus group DDP. (c) qRT-PCR method to detect the expression of Nrf2, SOD, CAT, and XDH mRNA in C2C12 myotube cells; RPS18 was used as an internal control. Data are shown as mean ± SD, *n* = 3/group; ^∗^*p* < 0.05, ^∗∗^*p* < 0.01, ^∗∗∗^*p* < 0.001 versus group DDP. (d) Nrf2 expression was detected by Western blotting: total Nrf2 expression using tubulin as the internal control and nuclear Nrf2 expression using Lamin A as the internal control; the relative expression level of protein was detected using ImageJ software. Tubulin and Lamin A were used as internal controls and normalized to the NC group. Data are shown as mean ± SD, *n* = 3/group; ^∗^*p* < 0.05, ^∗∗^*p* < 0.01, ^∗∗∗^*p* < 0.001 versus group DDP.

We investigated oxidative stress in C2C12 myotube cells 24 hours after exposure to the drug. As shown in Figures 6(a) and 6(b), ROS levels in the DDP group were significantly higher than those in the SLI groups. At the same time, SLI treatment resulted in a dose-dependent upregulation of mRNA expression of antioxidant enzymes, which decreased significantly after DDP treatment (Figure 6(c)). As shown in Figure 6(d), the expression of Nrf2 total protein and nuclear protein in C2C12 myotube cells treated with DDP decreased, further indicating that the cells were in a state of oxidative stress.

### 3.7. The Effects of SLI Can Be Blocked by the ERK Inhibitor Isofraxidin and the JNK Agonist 3′-Hydroxypterostilbene

**Figure 7:**
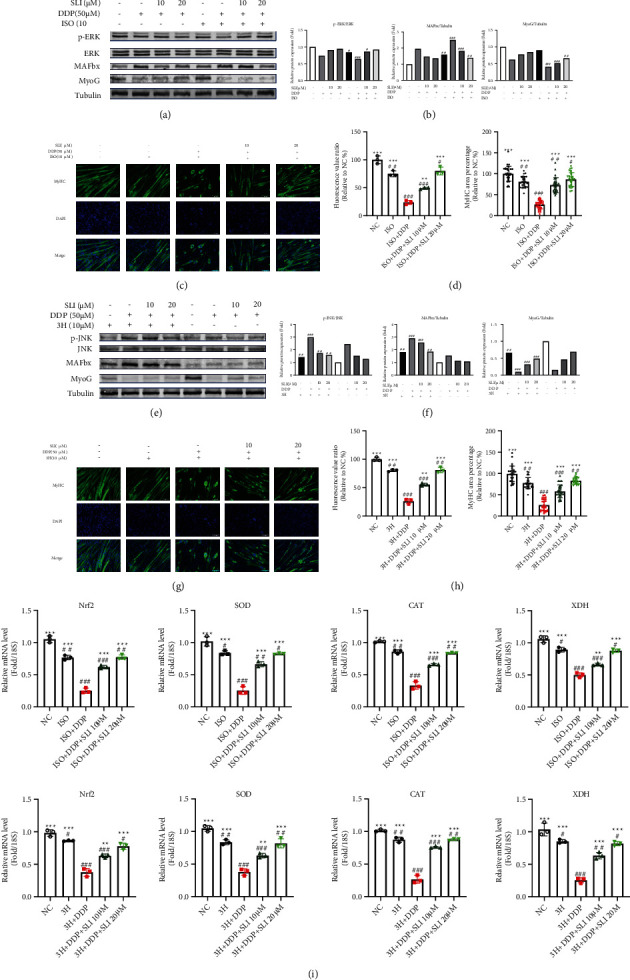
SLI plays a protective role through the ERK/FoxO and JNK/FoxO pathways. The ISO ERK inhibitor and the JNK agonist 3H can reduce the protective effect of SLI and aggravate oxidative stress. (a) Expression of MyoG, MAFbx, and ERK/FoxO signaling pathway proteins was detected by Western blotting, and tubulin was used as an internal control. (b) The relative expression levels of proteins were quantified using ImageJ software, and the relative expression levels of total proteins were normalized to tubulin and corrected to the untreated group. Data are shown as mean ± SD, *n* = 3/group; ^#^*p* < 0.05, ^##^*p* < 0.01, ^###^*p* < 0.001 versus group NC. (c) ISO attenuated the protective effect of SLI on DDP-induced differentiated C2C12 myotube cell atrophy (scale = 100 *μ*m). (d) The fusion index of C2C12 myotube cells differentiated by MyHC was measured by ImageJ software. Data are shown as mean ± SD, *n* = 3/group; ^∗∗^*p* < 0.01, ^∗∗∗^*p* < 0.001 versus group DDP; ^#^*p* < 0.05, ^##^*p* < 0.01, ^###^*p* < 0.001 versus group NC. (e) The expression of the MyoG, MAFbx, and JNK/FoxO signaling pathway proteins was detected by Western blotting, and tubulin was used as an internal control. (f) The relative expression of proteins was quantified using ImageJ software, and the relative expression levels of total proteins were normalized to tubulin and corrected to the untreated group. Data are shown as mean ± SD, *n* = 3/group; ^##^*p* < 0.01, ^###^*p* < 0.001 versus group NC. (g) 3H attenuated the protective effect of SLI on DDP-induced differentiated C2C12 myotube cell atrophy (scale = 100 *μ*m). (h) The fusion index of C2C12 myotube cells differentiated by MyHC was measured by ImageJ software. Data are shown as mean ± SD, *n* = 3/group; ^∗∗^*p* < 0.01, ^∗∗∗^*p* < 0.001 versus group DDP; ^##^*p* < 0.01, ^###^*p* < 0.001 versus group NC. (i) qRT-PCR method to detect the expression of Nrf2, SOD, CAT, and XDH mRNA in C2C12 myotube cells; RPS18 was used as an internal control. Data are shown as mean ± SD, *n* = 3/group; ^∗∗^*p* < 0.01, ^∗∗∗^*p* < 0.001 versus group DDP; ^#^*p* < 0.05, ^##^*p* < 0.01, ^###^*p* < 0.001 versus group NC.

In DDP-treated C2C12 myotube cells, SLI decreased the expression level of FoxO in the nucleus by interfering with the ERK/FoxO and the JNK/FoxO pathways, to inhibit the expression of MAFbx. The ERK inhibitor isofraxidin (ISO) inhibits ERK phosphorylation [51] and interfered with the effects of SLI on JNK/FoxO signaling. In this study, the expression of p-ERK was downregulated, the expression of the MAFbx protein was upregulated, and the expression of the MyoG protein was downregulated after using ISO alone (Figures 7(a) and 7(b)). Combined with DDP, ISO further inhibited the expression of MyHC and MyoG, upregulated the expression of MAFbx, and weakened the protective effects of SLI (Figures 7(a)–7(d)). The JNK agonist 3′-hydropterostilbene (3H) activates JNK phosphorylation [52] and blocked the inhibitory effect of SLI on the JNK/FoxO signal pathway. The use of 3H alone upregulated the expression of the p-JNK and MAFbx protein and downregulated the expression of the MyoG protein (Figures 7(e) and 7(f)). Combined with DDP, it furthered inhibit the expression of MyHC and MyoG, upregulated the expression of MAFbx, and weakened the protective effects of SLI (Figures 7(e)–7(h)). Furthermore, as shown in Figure 7(i), the effects of ISO and 3H negatively regulated the expression of mRNA of the antioxidant enzyme and weakened the effects of SLI. These findings suggested that SLI relied on the ERK/FoxO and the JNK/FoxO pathways to reduce oxidative stress and exert its antimuscle atrophy role.

## 4. Discussion

DDP is one of the most commonly used and effective chemotherapeutic agents for treating breast cancer, lung cancer, ovarian cancer, head and neck cancer, and bladder cancer. However, in the treatment of cancer, more serious adverse reactions, such as digestive system damage, hematopoietic system damage, kidney injury, cancer cachexia, and muscle atrophy, are often observed [53, 54]. These adverse reactions limit the clinical application of DDP and cause physical damage and psychological distress to patients. Therefore, reducing adverse reactions is very important for the clinical application of DDP. In this study, DDP not only treated tumors but also caused cancer cachexia. DDP-treated mice showed weight loss, reduced food intake, reduced grip strength, and tissue consumption. We used SLI to treat cachexia caused by DDP cancer, which produced beneficial effects.

DDP-induced cancer cachexia, likely due to the oxidative stress caused by DDP, leading to skeletal muscle atrophy is mediated by FOXO [11, 12] and results in cancer cachexia muscle atrophy. In this study, SLI treatment slowed DDP-induced weight loss and increased food intake and reduced muscle and fat mass loss, without affecting the antitumor effects of DDP. After treatment with DDP, the strength of mice decreased, the CAS of muscle fibers decreased, and type I decreased further, which allowed the body to regain strength more quickly, to compensate for the reduction of the overall strength of the body caused by the reduction of muscle mass [55]. Our results showed that SLI treatment alleviated the decrease in CAS in muscle fibers, maintained the content of two types of muscle fibers, and alleviated the decrease in muscle strength in mice induced by exposure to DDP.

It is well known that ROS coexists with hypoxia in the cancer microenvironment. The increase in ROS levels can directly lead to the activation of the ubiquitin proteasome system, resulting in skeletal muscle atrophy [43]. Under oxidative stress, ROS can upregulate the expression of inflammatory factors such as NF-*κ*B and IL-6. These inflammatory factors will lead not only to skeletal muscle atrophy but also to changes in skeletal muscle [56]. In addition, ROS-induced mitochondrial damage and autophagy also play an important role in skeletal muscle atrophy [20, 57]. An abnormal increase in ROS can cause damage to the body in many aspects. The transcription factor Nrf2 is a very important component in antioxidation mechanisms. Following Nrf2 nuclear translocation, it can positively regulate genes containing antioxidant response elements (ARE) in the promoter. The proteins encoded by these genes can participate in many protective reactions in the body, including antioxidant defense, protein degradation, cell growth, and DNA repair [24]. This may explain why the ROS content in the DDP-treated mice was significantly higher than that in other treatment groups in our study. After SLI treatment, abnormally elevated ROS in muscle tissue and C2C12 myotube cells were observed in the normal group. In addition, the treatment of SLI increased the expression of Nrf2 in the nucleus and the mRNA expression of SOD and cat and the content of GSH and GSSG in muscle tissue was also similar to that of the normal group, indicating that the body had restored the normal antioxidant levels in response to oxidative stress stimuli.

Furthermore, inhibition of Nrf2 leads to JNK/ERK-mediated apoptosis induced with oxidative stress [58]. JNK is often associated with apoptosis [56, 60]. ERK is related to cell growth, differentiation, and survival and to Nrf2 nuclear translocation [58, 61, 62]. When oxidative stress occurs, excessive ROS levels inhibit ERK phosphorylation and activate JNK phosphorylation, thus promoting cell apoptosis, which is related to skeletal muscle atrophy. Our experimental study also confirmed these findings: the ROS content in the DDP-treated group increased, ERK phosphorylation decreased, JNK phosphorylation increased, the expression of active caspase3 in the DDP-treated group increased, muscle was atrophied, and C2C12 myotube cells underwent apoptosis. Many studies have also shown that inhibition of ERK will lead to the failure of protein synthesis and activation of ERK can resist myotube atrophy and promote protein synthesis [61, 63, 64]. For example, eicosapentaenoic acid (EPA) and docosahexaenoic acid (DHA) inhibit the differentiation of C2C12 myotube cells by downregulating ERK [65]; lactic acid can increase the diameter of C2C12 myotube cells in a dose-dependent manner by activating ERK [66], while ferulic acid can induce the proliferation and differentiation of C2C12 myoblasts by activating ERK and Akt signals [67]. In addition, EGFR upstream of ERK is an epithelial polarity regulator. The EGFR signal will be enhanced during skeletal muscle regeneration. Activating EGFR-dependent polar pathways can promote the functional rescue of muscle cells and enhance muscle strength [68]. Our results also found that ERK phosphorylation in muscle tissue and C2C12 myotube cells in the DDP group was downregulated. At the same time, the expression of MyHC, MyoG, and MyoD related to myogenesis was downregulated, protein synthesis was reduced, the myotube diameter was reduced, and differentiation was blocked. In our study, SLI treatment increased ERK phosphorylation and increased expression of MyHC, MyoG, and MyoD, weakening DDP-induced atrophy. Other studies have shown that upregulation of JNK phosphorylation can promote MAFbx expression and induce muscle atrophy and cell death [69–71]. Therefore, inhibition of JNK phosphorylation can play a protective role. Curcumin reduces inflammatory damage to skeletal muscle cells by inhibiting the JNK/NF-*κ*B pathway [72] and Grifola frondosa promotes glucose uptake and growth of C2C12 myotube cells by downregulating JNK phosphorylation [73]. These results show that promoting ERK phosphorylation and inhibiting JNK phosphorylation can protect muscle tissue and C2C12 myotube cells.

In addition, inhibition of Nrf2 leads to JNK/ERK-mediated apoptosis related to oxidative stress [58]. JNK is often associated with apoptosis [56, 60]. ERK is related to cell growth, differentiation, survival and Nrf2 nuclear translocation [58, 61, 62]. When oxidative stress occurs, excessive ROS inhibits the phosphorylation of ERK and activates the phosphorylation of JNK, thus promoting cell apoptosis, which is related to skeletal muscle atrophy. Our experimental study also obtained such results: the content of ROS in DDP group increased, the phosphorylation of ERK decreased, the phosphorylation of JNK increased, the expression of active caspase3 in DDP group increased, the muscle was atrophied, and C2C12 myotube cells began apoptosis. Many research results also show that inhibition of ERK will lead to the failure of protein synthesis and the activation of ERK can resist myotube atrophy and promote protein synthesis [61, 63, 64]. For example, eicosapentaenoic acid (EPA) and docosahexaenoic acid (DHA) inhibit the differentiation of C2C12 myotube cells by downregulating ERK [65], lactic acid can increase the diameter of C2C12 myotube cells in a dose-dependent manner by activating ERK [66], and ferulic acid can induce the proliferation and differentiation of C2C12 myoblasts by activating ERK and Akt signals [67]. In addition, the epidermal growth factor receptor (EGFR) upstream of ERK is an epithelial polarity regulator. EGFR signal will be enhanced during skeletal muscle regeneration. Activating EGFR-dependent polar pathways can promote the functional rescue of muscle cells and enhance muscle strength [68]. Our results also found that ERK phosphorylation in muscle tissue and C2C12 myotube cells in the DDP group was downregulated. At the same time, the expression of MyHC, MyoG, and MyoD related to myogenesis was downregulated, protein synthesis was reduced, the myotube diameter was reduced, and differentiation was blocked. In our study, treatment with SLI increased ERK phosphorylation and upregulated expression of MyHC, MyoG ,and MyoD, weakening DDP-induced atrophy. Other studies have shown that the upregulation of JNK phosphorylation can promote the expression of MAFbx and induce muscle atrophy and cell death [69–71]. Therefore, inhibition of JNK phosphorylation can play a protective role: curcumin reduces the inflammatory damage of skeletal muscle cells by inhibiting the JNK/NF- *κ*B pathway [72], and *Grifola frondosa* promotes glucose uptake and growth of C2C12 myotube cells by downregulating JNK phosphorylation [73]. These results show that promoting ERK phosphorylation and inhibiting JNK phosphorylation can protect muscle tissue and C2C12 myotube cells.

The effects of ERK and JNK on muscle tissue and C2C12 myotube cells are related to the transcription factor FoxO3. FoxO3 is a member of the forkhead box (Fox) superfamily of transcription factors. FoxO3 is widely expressed in skeletal muscle and participates in a variety of cellular responses, such as autophagy, apoptosis, and stem cell homeostasis [74]. As a transcription factor, when FoxO3 translocates into the nucleus, it will induce the expression of muscle atrophy-related genes, eventually leading to protein degradation and skeletal muscle atrophy [75]. FoxO3 expression is negatively regulated by PI3K/Akt and MAPK/ERK, and positively regulated by the oxidative stress/JNK pathway [76]. Therefore, downregulation of ERK phosphorylation and upregulation of JNK phosphorylation can promote FoxO3 localization in the nucleus, resulting in atrophy and apoptosis. Our results also showed that FoxO3 expression in the nucleus increased in the DDP-treated group, while FoxO3 nuclear translocation decreased following SLI treatment. To demonstrate that JNK and ERK associated with oxidative stress are indeed related to muscle atrophy caused by DDP and the therapeutic effect of SLI, JNK phosphorylation agonists and ERK phosphorylation inhibitors were used in the study. Both decreased the mRNA expression of antioxidant-related enzymes and atrophied C2C12 myotube cells. These effects were alleviated after SLI intervention, suggesting that the therapeutic outcome associated with SLI is related to JNK and ERK, as well as oxidative stress.

**Figure 8:**
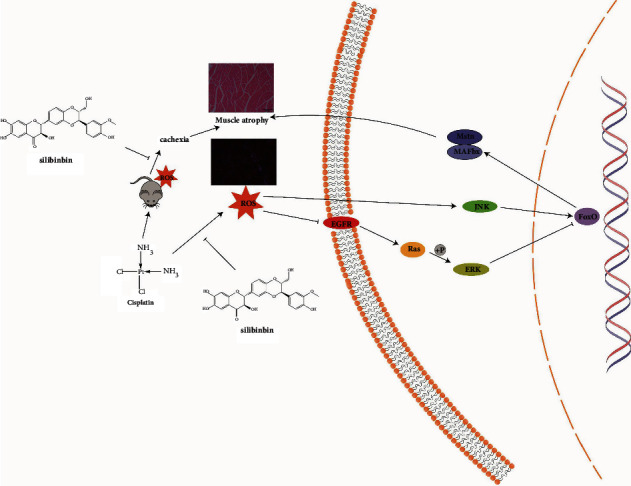
SLI can improve cachexia caused by DDP-induced oxidative stress. SLI reduces skeletal muscle atrophy by reducing oxidative stress, downregulating JNK phosphorylation, upregulating ERK phosphorylation, inhibiting FoxO entry into the nucleus, and downregulating the expression of MAFbx and Mstn.

In this study, SLI reduced DDP-induced skeletal muscle atrophy by regulating oxidative stress. We determined the content of silybin in muscle by LC-MS. The average content of muscle SLI in the DDP + SLI (L) group was 18.15 ± 3.56 ng/g (*n* = 5), and that in the DDP + SLI (H) group was 39.06 ± 6.67 ng/g (*n* = 5). We speculate that SLI enters the muscle to fight against DDP. The treatment of SLI prevents the cancer cachexia muscle atrophy caused by DDP. At the same time, the findings relative to the ROS content and Nrf2 and antioxidant enzyme expression showed a tendency to be normalized after SLI treatment; we speculate that SLI improves the antioxidant capacity of the body by regulating oxidative stress, to restore abnormally expressed ERK and JNK phosphorylation under oxidative stress; further, it prevents FoxO3 nuclear translocation into the nucleus and reduces protein decomposition and muscle atrophy. The proposed mechanism of SLI activity against DDP-induced skeletal muscle atrophy is shown in Figure 8.

SLI can protect skeletal muscle cells, reduce the damage caused by DDP-induced oxidative stress, and did not weaken the antitumor effects of DDP in vivo. SLI has been reported to have an antitumor effect [77, 78]. In this study, although additional SLI treatment with SLI reduced the tumor weight more than DDP treatment, there were no significant differences on tumor activity, which may be due to the short exposure to the drug. However, in this short period of time, SLI successfully antagonized skeletal muscle atrophy induced by DDP. This effect was associated with its antioxidant capacity. This characteristic of SLI is very important for the improvement of DDP-induced skeletal muscle atrophy and cachexia.

In our study, SLI protected skeletal muscle from DDP-induced damage. However, DDP may also lead to skeletal muscle atrophy by interfering with the insulin-like growth factor-1 (IGF-1)/PI3K/Akt pathway, autophagy, mitochondrial damage, upregulation of proinflammatory factors, calcium homeostasis, and lipid metabolism disorders. Whether the role of SLI is involved in these pathways and whether SLI combined with DDP can reduce side effects and increase antitumor effect need to be further evaluated.

## 5. Conclusion

SLI can improve the status of oxidative stress, improve the antioxidant capacity of the body, downregulate the expression of MAFbx and Mstn through the JNK/FOXO and ERK/FOXO signaling pathways, and reduce DDP-induced skeletal muscle atrophy. Although unable to influence antitumor activity, SLI can improve cachexia symptoms such as anorexia and weight loss induced by DDP.

## Figures and Tables

**Table 1:** Primers for RT-qPCR.

Primer	Sequence
Myh2 (MyHC IIA) (forward)	GCGACAGACACCTCCTTCAAGAAC
Myh2 (MyHC IIA) (reverse)	GTCCAGCCAGCCAGTGATGTTG
Myh4 (MyHC IIB) (forward)	TGATGCAGGCTGAGATCGAGGAG
Myh4 (MyHC IIB) (reverse)	TTGGTGTTGATGAGGCTGGTGTTC
Myh7 (MyHC I) (forward)	GCAAGACGGTGACTGTGAAGGAG
Myh7 (MyHC I) (reverse)	GGTTGACGGTGACGCAGAAGAG
MyoG (forward)	AGAGGAAGTCTGTGTCGGTGGAC
MyoG (reverse)	GTAGGCGCTCAATGTACTGGATGG
MyoD (forward)	CGTGGCAGCGAGCACTACAG
MyoD (reverse)	CGACACAGCCGCACTCTTCC
Fbxo32 (forward)	GTCGGCAAGTCTGTGCTGGTG
Fbxo32 (reverse)	AGGCAGGTCGGTGATCGTGAG
Mstn (forward)	CGATGAGCACTCCACGGAATCC
Mstn (reverse)	ACACTCTCCTGAGCAGTAATTGGC
Ub (forward)	CTCCTCCATCCACTCCTGACTCTG
Ub (reverse)	CACTGCCAGCATCTGAAGTTGTATTG
Nrf2 (forward)	CTGGGTTCAGTGACTCGGAAATGG
Nrf2 (reverse)	AGAATGTGCTGGCTGTGCTTTAGG
SOD (forward)	ACGCCACCGAGGAGAAGTACC
SOD (reverse)	GCTTGATAGCCTCCAGCAACTCTC
CAT (forward)	GGAGGCGGGAACCCAATAGGAG
Primer	Sequence
CAT (reverse)	TGTCAAAGTGTGCCATCTCGTCAG
XDH (forward)	AGAGCAGAGGACAGAGGTGTTCAG
XDH (reverse)	CCAATGGACGCCACGGACTTG
RPS18 (forward)	GTAACCCGTTGAACCCCATT
RPS18 (reverse)	CCATCCAATCGGTAGTAGCG

## Data Availability

The data used to support the findings of this study are available from the corresponding author upon request.

## Supplementary Materials

Supplementary Materials Figure S1: CCK8: SLI-treated C2C12 myotube cells and LLC cells for 24 and 48 hours, respectively. Figure S2: effects of 10 *μ*M and 20 *μ*M silybin on differentiated C2C12 myotube cells after 24 hours.
