# Supplementary material for: Silibinin Alleviates Muscle Atrophy Caused by Oxidative Stress Induced by Cisplatin through ERK/FoxO and JNK/FoxO Pathways
Source: Oxid Med Cell Longev. 2022 Jan 20;2022:5694223. doi: 10.1155/2022/5694223 (PMC8794676; doi:10.1155/2022/5694223)
Supplement: Supplementary Materials — Figure S1: CCK8: SLI-treated C2C12 myotube cells and LLC cells for 24 and 48 hours, respectively. Figure S2: effects of 10 μM and 20 μM silybin on differentiated C2C12 myotube cells after 24 hours. [file 5694223.f1.docx]

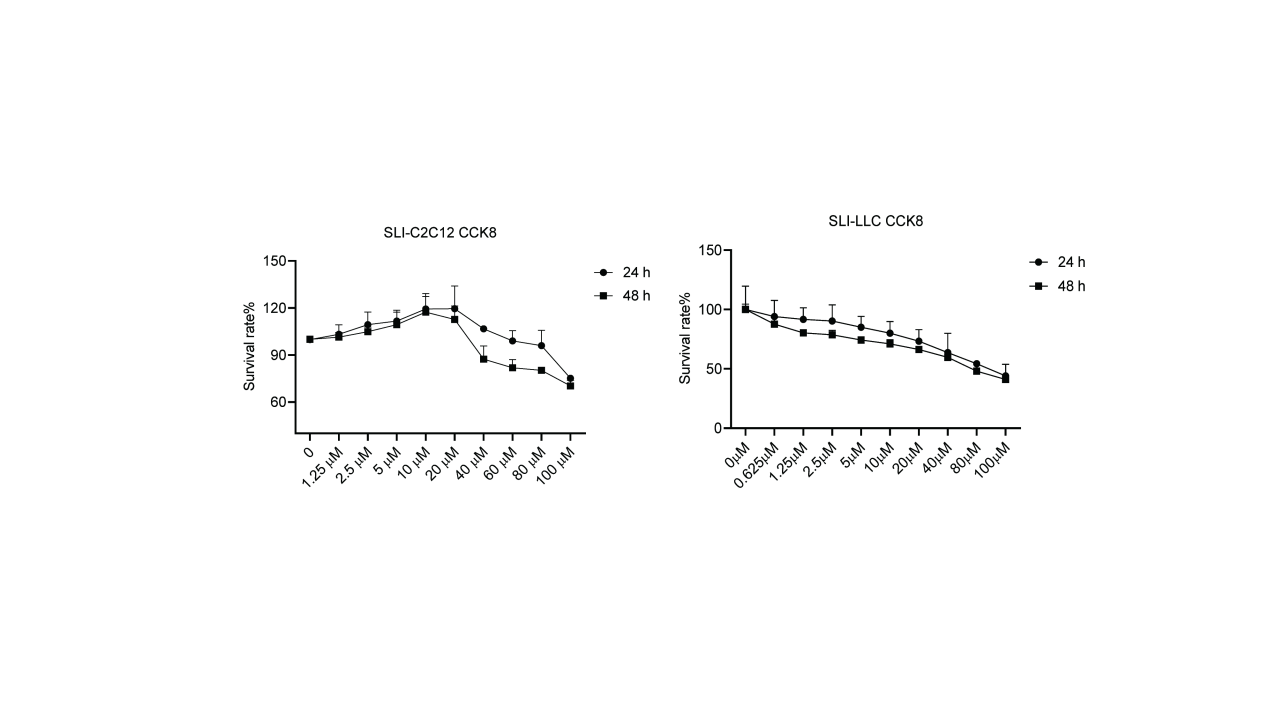


Figure S1: CCK8: SLI treated C2C12 myotube cells and LLC cells for 24 and 48 hours, respectively.


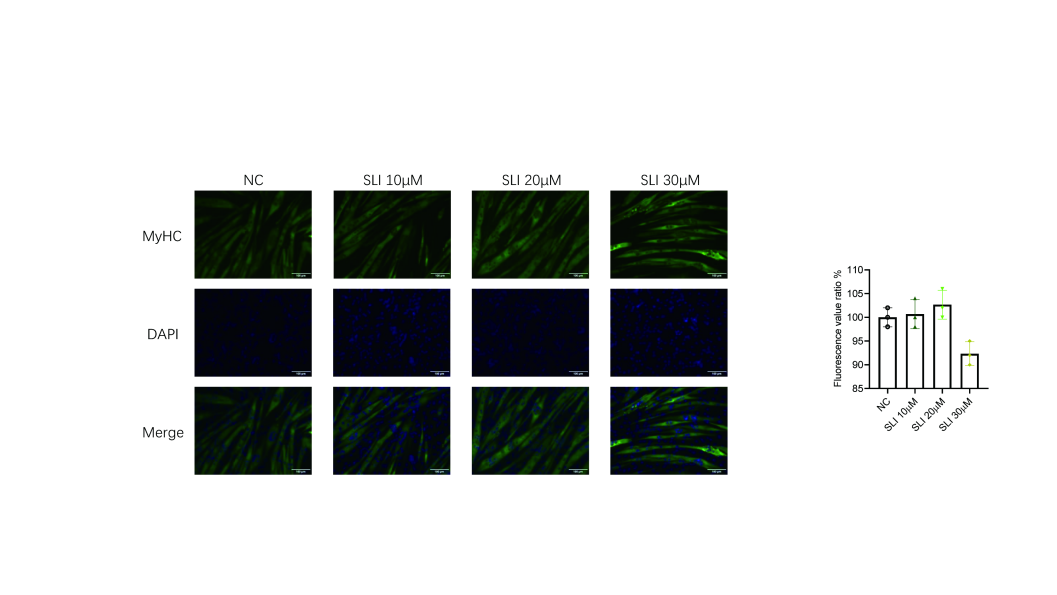


Figure S2: Effects of 10μM and 20μM silybin on differentiated C2C12 myotube cells after 24 hours.
